# 

**Published:** 1915-06-12

**Authors:** 


### CONTENTS.

PAGE

**Professional Procedure and Panel Patients** ... 217

**The Merriment of Mr. Stephen Coleridge** ... 218

**Hospital and Institutional News** ... ... ... 219

The Birthday Honours—Honours for Active Service—The Indian List—War Service Badges for Hospital Staffs—The Dismissal of Refractory Sanatorium Patients—Aid Raids on Hospitals—A German Matron—Travelling School Hospitals and Clinics—The South London Hospital for Women—Medical Women and Marriage—Tooting Home as a Military Hospital—General Medical Council's New Home—Ex-Hospital Clerk and Officer Sentenced—Hospital Memorial for Gretna Disaster—The Belgian Field Hospital Affair—Suggested Base Hospital for Bradford—The Nationality of a Guy's Medical Student—The Value of Small Functions—Railway Men and King Edward VII.'s Hospital, Cardiff—An Army Doctor Who Preferred Suicide to Promotion—German Suppression of the Belgian Red Cross—A Record Collection for a Record Work—The Rating of Hospitals Occupied by Wounded—Italy and the Drug Market.

**The Voluntary Hospitals and the Wounded** ... 225

The New Huts at St. Thomas's Hospital: The Problem of Additional Accommodation.

**Buildings Contemplated and in Progress** ... 226

**The Hospital Life of the Wounded** ... ... 227

Comradeship, Cheerfulness, and Cigarette-Smoking.

**My Commando Experiences in the Rebellion** 229

By a South African Medical Officer.

*[See over.]*

### CONTENTS—(*continued*).

| | PAGE |
|---|---|
| **Manchester Hospital War Work** | 230 |
| **A Medical Causerie** | 231 |
| **Round the World** | 232 |
| Fellow-Workers and the Roll of Honour. | |
| **War-Time at a Voluntary Hospital** | 233 |
| St. Thomas's Hospital as a Type. | |
| **The Gospel of Personal Service** | 236 |
| **The Voluntary Hospitals' Budget** | 238 |
| **St. Magnus the Martyr and Hospital Sunday** | 239 |
| **The Editor's Letter-Box** | 240 |
| Royal Devon and Exeter Hospital. | |
| The Seamen's Hospital. | |
| **Gifts and Bequests** | 240 |
| **Medical Missions and the War** | vi |
| **Chemistry and the War** | vi |
| **Personalia** | vi |
| **Retirement of a Veteran Administrator** | vi |
| **The Institutional Worker** (Suppl.) | 1 |
| Index System for Minute-Books : Answer to April Question. | |
| National Insurance Work : Answer to May Question. | |
| Question for June. | |
| Questions Invited. | |
| Enquiries and Answers. | |
| The Sick and In Need : Home Wanted—Paralysed Nurse—Convalescent Home for Wardmaid. | |
| Employment and Training : Short Training—Training for Health Visitor. | |
| War Matters : Massage for the Wounded. | |
| Miscellaneous : Artificial Limbs and Appliances—Gratifying Words. | |

